# Left Ventricular Hypertrophy and Insulin Resistance in Adults from an Urban Community in the Gambia: Cross-Sectional Study

**DOI:** 10.1371/journal.pone.0093606

**Published:** 2014-04-04

**Authors:** Bernard Cudjoe Nkum, Frank Botsi Micah, Theophilus C. Ankrah, Ousman Nyan

**Affiliations:** 1 Department of Medicine, School of Medical Sciences, Kwame Nkrumah University of Science and Technology, Kumasi, Ghana; 2 Department of Medicine, Royal Victoria Teaching Hospital, Banjul, The Gambia; CAEBi, Spain

## Abstract

**Objective:**

To determine the association between left ventricular hypertrophy and insulin resistance in Gambians.

**Design:**

Cross-sectional study.

**Setting:**

Outpatient clinics of Royal Victoria Teaching Hospital and Medical Research Council Laboratories in Banjul.

**Participants:**

Three hundred and sixteen consecutive patients were enrolled from outpatient clinics. The data of 275 participants (89 males) were included in the analysis with a mean (± standard deviation) age of 53.7 (±11.9) years.

**Interventions:**

A questionnaire was filled and anthropometric measurements were taken. 2-D guided M-mode echocardiography, standard 12-1ead electrocardiogram, fasting insulin and the oral glucose tolerance test were performed.

**Main Outcome Measures:**

The Penn formula was used to determine the left ventricular mass index, 125 g/m^2^ in males and 110 g/m^2^ in females as the cut-off for left ventricular hypertrophy. Using the fasting insulin and fasting glucose levels, the insulin resistance was estimated by the homeostatic model assessment formula. Logistic regression analysis was used to determine the association between left ventricular hypertrophy and insulin resistance.

**Results:**

The mean Penn left ventricular mass index was 119.5 (±54.3) and the prevalence of Penn left ventricular mass index left ventricular hypertrophy was 41%. The mean fasting glucose was 5.6 (±2.5) mmol/l, fasting insulin was 6.39 (±5.49) μU/ml and insulin resistance was 1.58 (±1.45). There was no association between Penn left ventricular mass index left ventricular hypertrophy and log of insulin resistance in univariate (OR = 0.98, 95% CI = 0.80 – 1.19, p = 0.819) and multivariate logistic regression (OR = 0.93, 95% CI = 0.76–1.15, p = 0.516) analysis.

**Conclusion:**

No association was found in this study between left ventricular hypertrophy and insulin resistance in Gambians and this does not support the suggestion that insulin is an independent determinant of left ventricular hypertrophy in hypertensives.

## Introduction

Left ventricular hypertrophy (LVH) and insulin resistance (IR) are both strong adverse factors for cardiovascular disease. The co-existence of LVH and IR is a clinical finding, which must be taken seriously, even in the absence of blood pressure levels above the usual limits for initiating drug therapy because it is a predictor of adverse risk for mortality and morbidity.

The association of hypertension and IR has been studied extensively as part of the metabolic syndrome [1]. The association between hypertension and LVH is also well established with hypertension being one of the leading causes of LVH [2]–[4]. Answers to whether IR plays any role in the pathogenesis of LVH from the various studies have been variable, with some showing a clear relationship while others have demonstrated no association between IR and LVH.

In a study of 40, otherwise healthy, non-diabetic, normotensive obese subjects Sasson et al demonstrated that IR was strongly associated with LVH and that this association was independent of blood pressure and body mass index (BMI) [5]. Lind et al., found LVH to be closely associated with IR and in multiple regression analysis IR accounted for 47% of the variability of left ventricular mass [6].

IR was found to have no influence on left ventricular mass index in a study of 29 non-obese hypertensive patients [7]. Another study of 50 non-diabetic participants revealed that after controlling for blood pressure and BMI, insulin concentration, secretion and action was not an independent determinant of LVH [8].

The prevalence and relevance LVH and IR in Gambians are unknown. The aim and objective of this study are to determine the association between LVH and IR in Gambians.

## Materials and Methods

### Ethics Statement

The study was approved by The Gambia Government/MRC Ethical Committee. All the participants signed or thumb printed an informed consent form after careful consideration and explanation.

This was a cross sectional study conducted at the Royal Victoria Teaching Hospital, Banjul and the Medical Research Council (MRC) Laboratories, Fajara, The Gambia. Patients with hypertension attending the Royal Victoria Teaching Hospital hypertension clinic were recruited consecutively. The non-hypertensive patients were recruited from the Gate Clinic of the MRC Laboratories, Fajara. These were patients who reported with minor infections and were not diabetic and had no cardiovascular disease. The recruitment of participants was conducted from January to May 2000.

Patients with morbid obesity (BMI>35 kg/m^2^), systemic or metabolic diseases, severe inter-current illnesses; known diabetes mellitus, cardiovascular disease (excluding hypertension) or labile hypertension (labile hypertension is when the blood pressure is sometimes above or sometimes below 140/90 mmHg), [9] were excluded from the study. Patients who were not known diabetes mellitus patients but who were found to be so after oral glucose tolerance test (OGTT) were included.

A questionnaire was administered by a field worker using the appropriate local language and a physical examination was done by one physician. The weight of participants was measured (to the nearest 0.1 kg) on electric scales (Secca r 770, CMS London), with subjects wearing light clothes and without footwear. The height was measured to the nearest 0.5 cm without footwear or head gear using standardised stadiometers. Hip and waist circumferences were measured using a plastic tape measure and recorded to the nearest 0.5 cm. The blood pressure was measured on the left arm using digital blood pressure machines (Omron r HOM – 705 CP, Japan) [10] and these were calibrated to the standard mercury sphygmomanometer every two weeks. Three readings were taken and the mean of the later two readings was used in the analysis [11].

A 3.5 MHz transducer on Hitachi EUB – 405 ultrasound scanner was used in performing 2-dimensional M-mode guided echocardiography on participants. The subjects were studied most commonly in the left lateral or partial left lateral position. The posterior left ventricular wall thickness was measured from the endocardial echo to the edge of the acoustic epicardial echo while the inter-ventricular septum was measured between its endocardial echoes [12].

The left ventricular end-diastolic diameter (LVEDD) was measured from the leading edge of the anterior endocardium to the leading edge of the posterior wall endocardium at end-diastole. The left ventricular end-systolic diameter (LVESD) was measured from inner edge of the anterior endocardium to the inner edge of the posterior wall endocardium of the left ventricle at end-systole. Polaroid paper were used to record all the distances, time and heart rate during the procedure.

An OGTT was carried out on participants during which, insulin levels were measured. The OGTT was performed using 75 g anhydrous glucose in 300–350 ml of water. A Haemocue analyser (Haemocue AB, Sweden) was used to immediately determine glucose level on fasting, 30 min and 120 min samples. The detailed results of the OGTT are presented in another article which is in print. However the information was used in classifying participants as DM in this study. Insulin level of a fasting sample was measured in the MRC NCD Laboratory with the Abbott IMx Immunoassay Analyser, which utilises a Micro-particle Enzyme Immunoassay (MEIA).

Using the fasting insulin and fasting glucose levels, the IR was estimated by the homeostatic model assessment (HOMA) formula.

The following definitions were adopted for this study.

1. Hypertension is systolic blood pressure≥140 and/or diastolic blood pressure≥90.mmHg in subjects who are not taking antihypertensive medication.

2. Overall Obesity is Body Mass Index (BMI)≥30 kg/m^2^.

3. Central Obesity or High Waist Hip Ratio (WHR) is WHR>0.9 for males and>0.8 for females.

4. Diabetes. World Health Organisation Study Group on Diabetes Mellitus (1998). Fasting venous blood glucose>7.0 mmol/L and or 2 h post glucose capillary whole blood≥11.1 mmol/L [13].

5. Insulin Resistance (IR)  =  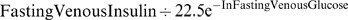 (The homeostasis model assessment formula).

6. Echocardiographic LVH Criteria

Penn convention/Formula: Left ventricular mass (PLVM) (g)

 =  1.04 {(IVSTD + LVPWTD + LVEDD) 3 – LVEDD3} – 13.6

Penn left ventricular mass indexed to body surface area (PLVMI) (g/m^2^)

Body Surface Area (m^2^)  =  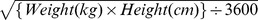


Penn left ventricular mass index (PLVMI)  =  PLVM/BSA

Penn left ventricular echocardiographic LVH (PLVMILVH) defined by:

PLVMI>125 g/m^2^ in males and PLVMI>110 g/m^2^ in females [14].

7. Smoking was defined as “ever smoked” as compared to non-smoking which was defined as “never smoked”.

The data was analysed using Stata version 8.0. The mean and standard deviation (sd) were used for continuous variables, and were compared using standard t-test. Discrete variables were analysed using Pearson chi squared test. The results of OGTT and IR were not normally distributed so a logarithmic transformation was done and this was used in all further analysis. Univariate and multivariate linear regression was used to analyse the association between log of IR and variables such as sex, smoking, hypertension, DM, BMI≥30, high WHR, antihypertensive treatment, age, BMI, WHR, SBP, DBP and fasting glucose. The association between PLVMILVH and log of IR was analysed using univariate and multivariate logistic regression controlling for various confounding variables. P-values of less than 0.050 were taken as statistically significant.

## Results

Two hundred and eight consecutive patients (70 males) with hypertension on treatment and 108 non-hypertensive patients (39 males) were enrolled from outpatient clinics for our initial study [15]. However the data of 275 participants (89 males) were included in this analysis and results. Those excluded had missing variables especially of IR or echocardiographic LVH.

The mean (± sd) age of participants was 53.7 (±11.9) years and the age range was 27–85 (Tables 1 and 2). The males were older than the females (p = 0.051). Weight was similar in both males and females but because the males were significantly taller their BMI was significantly lower than the females (p<0.0001). Prevalence of general obesity was 26% and this was significantly higher in the females (p<0.0001). The prevalence of central obesity was much higher (73%) and this was also higher in the females (p<0.0001). Mean systolic blood pressure (SBP) (136 mmHg), mean diastolic blood pressure (DBP) (83 mmHg), prevalence of hypertension (65%) and undiagnosed diabetes mellitus (DM) (15%) were similar in both the males and females. Smoking was more common in the male participants (p<0.001).

**Table 1:** The clinical characteristics of participants by sex.

	Male (n = 89)	Female (n = 186)	All (n = 275)	P
	Mean (SD)	Mean (SD)	Mean (SD)	(t test)
Age (years)	55.7 (10.0)	52.7 (12.6)	53.7 (11.9)	0.051
Weight (kg)	70.8 (15.4)	71.1 (15.8)	70.8 (15.6)	0.591
Height (m)	1.70 (0.07)	1.61 (0.06)	1.64 (0.08)	<0.0001
BMI (kg/m^2^)	24.1 (5.2)	27.5 (6.1)	26.4 (6.0)	<0.0001
WHR	0.89 (0.06)	0.88 (0.07)	0.88 (0.06)	0.066
SBP (mmHg)	140 (26)	134 (27)	136 (27)	0.134
DBP (mmHg)	83 (13)	83 (14)	83 (14)	0.678

t  =  Two sample t test with equal variance.

P  =  Statistical significance of difference.

n  =  Number.

SD  =  Standard deviation.

BMI  =  Body Mass Index BMI ≥ 30 (kg/m^2^)  =  General obesity.

WHR  =  Waist hip ratio  =  Central obesity.

SBP  =  Systolic Blood Pressure.

DBP  =  Diastolic Blood Pressure.

**Table 2:** The clinical characteristics of participants by sex.

	Male (n = 89)	Female (n = 186)	All (n = 275)	P
	Number (%)	Number (%)	Number (%)	(χ ^2^ test)
Age Range (yr)	32–80	27–85	27–85	
Smoking	46 (51.7)	15 (8.1)	61 (22.2)	< 0.001
Hypertension	60 (67.4)	119 (64.0)	179 (65.1)	0.576
Diabetes	12 (13.5)	29 (15.6)	41 (14.9)	0.646
BMI≥30	9 (10.1)	61 (32.8)	70 (25.5)	<0.001
HIGH WHR	37 (41.6)	163 (87.6)	200 (72.7)	<0.001
Antihypertensive Treatment	55 (61.8)	111 (59.7)	166 (60.4)	0.737

χ ^2^  =  Pearson chi squared test.

P  =  statistical significance of difference.

n  =  Number.

BMI≥30 (kg/m^2^)  =  General obesity.

WHR  =  Waist hip ratio  =  Central obesity.

HIGH WHR  =  WHR>0.9 for males and>0.8 for females.

There were 166 (60.4%) participants (55 males) on antihypertensive drugs and all of them were hypertension patients. However 13 hypertensives were not on drug treatment and none of the nonhypertensives were on antihypertensive treatment. There were 28 participants on a combination of 3 drugs, 94 on a 2 drug combination and 44 on monotherapy. The majority (137 participants) were on bendrofluazide and this was followed by methyldopa, propranolol, nifedipine, hydralazine, hydrochlorothiazide and atenolol in that order. There was a patient each on amlodipine and captopril.

The echocardiographic characteristics of participants were all significantly higher in the males than the females except LVEDD which was similar in both sexes (Table 3). The mean (± sd) PLVMI was 135.3 (±56.2) for the males, 111.9 (±51.8) for the females and 119.5 (±54.3) for all the participants. The prevalence of echocardiographic LVH, PLVMILVH was 49% in males, 37% in females and 41% for both males and females.

**Table 3:** The echocardiographic characteristics of participants by sex.

	Male (n = 89)	Female (n = 186)	All (n = 275)	P
	Mean (SD)	Mean (SD)	Mean (SD)	(t test)
LVPWTD (mm)	1.2 (0.3)	1.1 (0.3)	1.1 (0.3)	0.003
IVSTD (mm)	1.3 (0.4)	1.2 (0.3)	1.2 (0.4)	0.002
LVEDD (mm)	4.3 (0.6)	4.2 (0.7)	4.2 (0.7)	0.121
LVESD (mm)	28.6 (6.4)	26.7 (6.8)	27.3 (6.8)	0.032
PLVM (g)	245.5 (102.1)	198.0 (93.0)	213.4 (98.4)	<0.001
PLVMI (g/m^2^)	135.3 (56.2)	111.9 (51.8)	119.5 (54.3)	<0.001
				
*PLVMILVH (%)	44 (49.4)	69 (37.1)	113 (41.1)	0.052 ((χ ^2^)
				

t  =  Two sample t test with equal variance.

χ ^2^  =  Pearson chi squared test.

P  =  statistical significance of difference.

n  =  Number.

SD  =  Standard deviation.

* Number.

There were no statistically significant sex differences in the biochemical characteristics of the participants (Table 4). The mean (± sd) fasting glucose was 5.6 (±2.5) mmol/l, the mean fasting insulin was 6.39 (±5.49) μU/ml and the mean IR (HOMA score) was 1.58 (±1.45).

**Table 4:** The biochemical characteristics of participants by sex.

	Male (n = 89)	Female (n = 186)	All (n = 275)	P
	Mean (SD)	Mean (SD)	Mean (SD)	(t test)
Fasting Glucose (mmol/l)	5.9 (3.4)	5.5 (1.8)	5.6 (2.5)	0.240
Fasting Insulin (μU/ml)	6.04 (5.34)	6.55 (5.57)	6.39 (5.49)	0.470
Insulin Resistance (HOMA score)	1.50 (1.42)	1.62 (1.46)	1.58 (1.45)	0.516
				

t  =  Two sample t test with equal variance.

P  =  statistical significance of difference.

n  =  Number.

SD  =  Standard deviation.

In univariate logistic regression analysis with PLVMILVH as the outcome variable there were significant association between PLVMILVH and sex (p = 0.052), hypertension (p = 0.008), antihypertension treatment (p = 0.028), age (p = 0.002), WHR (p = 0.021) and SBP (p = 0.001)(Table 5). However there was no association between PLVMILVH and DM (p = 0.771), smoking (p = 0.082), log of fasting glucose (p = 0.885), high WHR (p = 0.382), BMI (p = 0.284), DBP (p = 0.056) and BMI > 30 (p = 0.234).

**Table 5:** Univariate analysis of variables with PVLMILVH as the outcome variable.

	OR	CI	P
Sex	0.60	0.36–1.00	0.052
Smoking	1.66	0.94–2.95	0.082
Hypertension	2.04	1.21–3.45	0.008
Diabetes	0.90	0.46–1.78	0.771
BMI≥30	1.39	0.81–2.41	0.234
HIGH WHR	0.79	0.46–1.35	0.382
Age	1.03	1.01–1.06	0.002
Weight	1.01	1.00–1.03	0.164
Height	1.83	0.09–35.36	0.689
BMI	1.02	0.98–1.06	0.284
WHR	1.97	1.01–2.02	0.021
SBP	1.02	1.01–1.03	0.001
DBP	1.02	1.00–1.04	0.056
Log of Fasting Glucose	1.07	0.42–2.75	0.885
Log of Insulin Resistance	0.98	0.80–1.19	0.819
Antihypertensive Treatment	0.57	0.34–0.94	0.028

P  =  Statistical test of significance of association.

CI  =  Confidence Interval.

OR  =  Odds Ratio.

BMI  =  Body Mass Index BMI≥30 (kg/m^2^)  =  General obesity.

WHR  =  Waist hip ratio  =  Central obesity.

SBP  =  Systolic Blood Pressure.

DBP  =  Diastolic Blood Pressure.

HIGH WHR  =  WHR>0.9 for males and >0.8 for females.

With log of IR as the outcome variable there were significant association between log of IR and DM (p<0.001), high WHR (p = 0.038), WHR (p = 0.006), BMI (p<0.001) and log of fasting glucose (p<0.001) (Table 6). There was no association between log of IR and sex (p = 0.667), smoking (p = 0.229), hypertension (p = 0.272), antihypertension treatment (p = 0.202), BMI > 30 (p = 0.080), age (p = 0.971), SBP (p = 0.723) and DBP (p = 0.999).

**Table 6:** Univariate analysis of variables with log of Insulin Resistance as the outcome variable.

	R	CI	P
Sex	0.07	−0.24–0.37	0.667
Smoking	−0.21	−0.55–0.13	0.229
Hypertension	0.17	−0.13–0.46	0.272
Diabetes	0.72	0.33–1.11	<0.001
BMI≥30	0.29	−0.04–0.62	0.080
HIGH WHR	0.34	0.02–0.65	0.038
Age	0.0002	−0.01–0.10	0.971
Weight	0.02	0.01–0.03	<0.001
Height	0.64	−1.11–2.40	0.472
BMI	0.05	0.02–0.07	<0.001
WHR	3.08	0.91–5.26	0.006
SBP	−0.001	−0.01–0.004	0.723
DBP	−0.001	−0.01–0.01	0.999
Log of Fasting Glucose	0.98	0.43–1.53	<0.001
PLVMILVH	0.03	−0. 32–0.26	0.820
Antihypertensive Treatment	−0.19	−0.48–0.10	0.202

P  =  Statistical test of significance of association.

CI  =  Confidence Interval.

R  =  Regression Coefficient.

BMI  =  Body Mass Index BMI≥30 (kg/m^2^)  =  General obesity.

WHR  =  Waist hip ratio  =  Central obesity.

SBP  =  Systolic Blood Pressure.

DBP  =  Diastolic Blood Pressure.

HIGH WHR  =  WHR>0.9 for males and >0.8 for females.

More importantly there was no association between PLVMILVH and log of IR in univariate logistic regression analysis (OR = 0.98, p = 0.819) (Table 5).

From the results of the univariate analysis WHR was the only variable which was associated with both PLVMILVH and log of IR. Therefore in a multiple logistic regression analysis we modeled with PLVMILVH as the outcome variable and WHR and log of IR as the explanatory variables. There was no significant association between PLVMILVH and log of IR (OR = 0.93, p = 0.516). WHR was however still associated with both PLVMILVH and log of IR (OR = 2.01, p = 0.017). There was still no significant association between PLVMILVH and log of IR in other models with PLVMILVH as the outcome variable and controlling for different variables. In further multivariate analysis with PLVMILVH as the outcome variable and controlling for various variables which were associated PLVMILVH in univariate analysis, the following variables were found to be associated with PLVMILVH, age (OR = 1.03, p = 0.018) and SBP (OR = 1.01, p = 0.006). When multiple regression analysis was done with log of IR as the outcome variable and the variable which were associated with log of IR in univariate analysis were included, BMI (r = 0.05, p<0.001) and log of fasting glucose (r = 0.98, p<0.001) were found to be the variables which were significantly associated with log of IR.

Further subgroup univariate logistic regression analysis with PLVMILVH as the outcome variable and log of IR as the explanatory variables was carried out for the various categorical variables. There was no association between PLVMILVH and log of IR in males, females, smokers, non-smokers, hypertensives, non-hypertensives, DM, non-DM, BMI ≥ 30, BMI < 30, high WHR and normal WHR (Table 7).

**Table 7:** Multivariate analysis of variables with PVLMILVH as the outcome variable and log of Insulin Resistance as the explanatory variable.

	OR	CI	P
Male	1.14	0.80–1.63	0.475
Female	0.91	0.71–1.17	0.471
Smoking	1.07	0.74–1.55	0.735
No smoking	0.96	0.75–1.22	0.732
Hypertension	0.89	0.71–1.13	0.335
No hypertension	1.23	0.79–1.93	0.363
Diabetes	1.01	0.54–1.90	0.981
No diabetes	0.98	0.79–1.22	0.851
BMI≥30	1.33	0.89–1.98	0.160
BMI<30	0.85	0.66–1.08	0.174
HIGH WHR	0.95	0.75–1.20	0.645
Normal WHR	1.11	0.75–1.65	0.602
Antihypertensive Treatment	0.94	0.74–1.19	0.603
No AntihypertensiveTreatment	1.01	0.70–1.47	0.950

P  =  Statistical test of significance of association.

CI  =  Confidence Interval.

R  =  Regression Coefficient.

BMI  =  Body Mass Index BMI≥30 (kg/m^2^)  =  General obesity.

WHR  =  Waist hip ratio  =  Central obesity.

HIGH WHR  =  WHR>0.9 for males and >0.8 for females.

## Discussion

In this study the relationship between LVH and IR was investigated using fasting blood glucose, fasting insulin and the HOMA formula for estimating IR and PLVMI. There was no statistical significant association between PLVMILVH and IR in these participants in univariate and multivariate regression analysis after controlling for various variables. Also in subgroup analysis using univariate logistic regression analysis there was no significant association between these two variables. Therefore, IR may not play a role in the development of LVH in this population of Gambians.

The most significant risk factors for LVH were the older the age of the subject and the higher the SBP rather than IR. On the other hand the significant risk factors for IR were high BMI and high fasting blood glucose. WHR which was a common significant variable to both LVH and IR was still significant in the multiple regression model but after adjusting for this variable there was still no significant association between LVH and IR.

Several mechanisms have been proposed to explain why there should be a direct relationship between IR and LVH. To date the best indirect evidence is the fact that angiotensin II receptor blockers correct both IR and LVH [16]. Angiotensin II type 1 receptors activity and numbers are up-regulated by the presence of IR [17]. LVH on the other hand is promoted by the mitogenic effect of angiotensin II on the angiotensin II type 1 receptors on smooth muscle cell of blood vessels. This eventually leads to hypertrophy of arterial wall and increased vascular resistance [18]. The effect of angiotensin II via angiotensin II type 1 receptors on the myocytes of the heart also directly promotes LVH [19]. This stimulation of LVH may partly be explained by an increased oxidative stress of the myocardium caused by angiotensin II [20].

Other direct and indirect mechanisms have been proposed for the contribution of IR in the development of LVH. These include the disordered and increased re-adsorption of sodium in the kidneys [21], [22], the increased activation of the sympathetic nervous system [23] and the production of insulin growth factor-1 [24]. Others are the direct action of increased levels of insulin on the myocytes of the heart resulting in cardiac hypertrophy and remodeling, the promotion of the growth of vascular smooth muscle cells and lipotoxicity. These mechanisms also stimulate LVH further through the promotion of arteriosclerosis and increased arterial wall stiffness [22], [25]. IR results in compensatory hyperinsulinemia which is considered to be a crucial factor in the development of the metabolic syndrome [26], [27].

The association between LVH and IR from the published literature has been variable. There have been several studies reporting a positive relationship while there are not too few papers which have established that there is no relationship between LVH and IR. Significant associations between IR and LVH have been demonstrated in hypertensive patients as well as obese subjects [5], [28]–[30]. The Framingham Heart Study showed a positive correlation of IR to LVH in female participants, but not in male subjects. However this correlation in females was attenuated after adjusting for BMI [31]. Our study did not show a significant association between LVH and IR before and after adjusting for hypertension, sex, general or central obesity. Further there was no significant association between LVH and IR in males nor females, hypertensives nor obese particiapants.

Other studies however have shown only weak association between LVH and IR [32] or no correlation at all after adjustments for other variables [7], [8], [33], [34]. In a study of 107 males aged 50 years and over, no relationships were observed between IR and LVH [35]. Top *et al* demonstrated in 70 diabetic patients that there was no statistically significant correlation between LVH and HOMA derived IR [36]. In our study we excluded all previously diagnosed DM patients but included 41 previously undiagnosed DM patients who were diagnosed from OGTT. Among this subgroup there was no significant association between LVH and IR, a finding similar to the Top *et al* study. IR and fasting insulin has also been shown not to be associated with LVH in healthy people, independent of obesity. This was in a cross-sectional relational study carried out in 153 healthy subjects, comprising 76 men and 77 women who were normotensive, had a normal oral glucose tolerance test, no cardiovascular disease and none were on any medication [37]. Sherif *et al* investigated the relationship between left ventricular mass index (using cube function indexed to height in 52 premenopausal African American women) and cardiovascular risk factors [38]. They used the oral glucose tolerance test and the euglycaemic clamp. Left ventricular mass index was not associated with IR [38]. These findings are similar to our study where there was no significant association between LVH and IR before and after controlling for diabetes, fasting glucose and BMI.

This study is one of the few cardiovascular studies which have been undertaken in The Gambia. To date there has not been any published data on LVH nor IR from this small West African country. This is the major strength of this pioneering study. The finding of no association between LVH and IR therefore raises the question as to whether this is an observation peculiar to this community or part of the world. The main weakness of the study was the fact that it was a hospital based study which was fraught with various biases including selection and proximity biases. Another potential limitation of this study was the fact that the insulin levels were measured only in the fasting state but not after the glucose load was administered. Some studies have demonstrated a positive association between LVH and postload insulin levels [30]. Further as a cross sectional study the findings needs to be further confirmed by bigger studies of different and better study designs such as longitudinal cohort studies. Consequently there is the urgent need for more cardiovascular studies generally in The Gambia and specifically to explore the relationship between LVH and IR. These surveys should be larger community based studies as well as in participants with specific risk factors like obesity, metabolic syndrome, hypertension and DM.

## Conclusion

There was no significant association between echocardiographic LVH and IR in this population of Gambians. Our findings do not support the suggestion that insulin is an independent determinant of LVH in hypertensives.
